# Pitfalls encountered while projecting the future Israeli anesthesiologist workforce: lessons from the Israel anesthesiologist workforce study

**DOI:** 10.1186/s13584-026-00780-1

**Published:** 2026-08-28

**Authors:** Ariel Wimpfheimer, Yehuda Ginosar, Daniela Quesada, Shai Fein, Charles Weissman, The Israel Anesthesiology Workforce Study Group, Haled Abd-Al-Halim, Hakeem Abu-Rais, Chaim Berkenstadt, Ilya Chernoy, Maruan Armaly, Yaakov Duvdivani, Leonid Eidelman, Shai Fein, Brian Fredman, Yulia Gadulov, Zeev Goldik, Yaakov Gozal, Zoya Haituv, Alex Izakson, Yaakov Katz, Idit Matot, Noam Mubada, Reuven Pizov, Aeyal Raz, Gefen Revaz Assuta, Igor Reznikof, Nogzar Rigzny, Michael Rudin, Vladimir Rukinglass, Albert Sabatnitzki, Eran Segal, Eric Siton, Mustafa Somri, Riad Tome, Jacob Turban, Nathan Weksler, Dafna Wilner, Yossi Witchelevsky, Alex Zlotnik

**Affiliations:** 1 Faculty of Medicine, Hebrew University of Jerusalem, Jerusalem, Israel; 2 Braun School of Public Health, Hebrew University of Jerusalem, Jerusalem, Israel; 3 Department of Anesthesiology, Critical Care Medicine and Pain Management, Hadassah-Hebrew University Medical Center, Jerusalem, Israel; 4 Department of Anesthesiology and Operating Rooms, Bellinson Hospital, Rabin Medical Center, Petach Tikva, Jerusalem, Israel; 5 Hospital Administration, Hadassah-Hebrew University Medical Center, Kiryat Hadassah, POB 12000, Jerusalem, 91120 Israel

**Keywords:** Anesthesiology, Anesthesiologists, Physician workforce, Forecasting methods, Projections

## Abstract

**Background:**

The smooth and efficient operation of a country’s healthcare system is highly dependent on the size and specialty composition of its physician workforce. Therefore, it is incumbent upon a country’s healthcare leadership to continually examine the workforce situation in each medical specialty, especially critical specialties like anesthesiology, so as to detect issues that could lead to shortfalls and surpluses and then apply effective corrective actions. The aim was to assess the accuracy of the different predictive methods and identify possible ways of improving their predictive capabilities. Our assumption was that the accuracy of workforce predictions is primarily affected by unforeseen and disruptive events.

**Methods:**

Cross-sectional surveys of the Israeli anesthesiology workforce were performed in 2005 (*n* = 711 anesthesiologists) and 2021 (*n* = 1330 anesthesiologists). This permitted comparing needs and demand-based workforce forecasts made in 2005 with the actual 2021 workforce. Reasons for discrepancies between the 2005 forecasts and the reality in 2021 were sought.

**Results:**

The 2005 needs-based projections underestimated the actual increase in anesthesiologists because the population grew at a rate faster than that estimated in 2005. Furthermore, there was an increase in the proportion of the population > 65 years old, an increase in life expectancy and a higher birth rate requiring a greater number of caesarean sections and epidural catheter insertions. Moreover, the 2011 union contract between the government and the Israel Medical Association increased anesthesiologist vacation time by two weeks per year. The demand-based projection underestimations were attributed to the significant increase in anesthetics performed outside operating rooms, such as, in cardiac catheterization laboratories and invasive radiology and endoscopy suites. Comparing anesthesiologist workforce projections made in 2005 to the workforce realities 16 years later revealed that many unpredictable (greater population growth) and disruptive (union contract) factors that ensued during that time period led to underestimating future workforce needs.

**Conclusions:**

Forecasting the future Israeli physician anesthesiologist workforce is an endeavor fraught with unpredictable challenges. To facilitate frequently updated forecasts of future workforce needs, healthcare systems require robust, long-term forecasting capabilities that use near real-time data obtained from the ubiquitous electronic medical and hospital record systems. These data should then be entered into sophisticated data analytic tools, rather than simple needs and demand-based methods. Such up-to-date forecasts should help healthcare system leaders and policy makers make more informed workforce decisions.

## Background

The smooth and efficient operation of a country’s healthcare system is significantly dependent on the size and specialty composition of its physician workforce. Imbalances in the number of physicians in any given specialty is undesirable, with inadequate numbers leading to healthcare shortages and excessive waiting times, while a surplus can result in the overutilization of some medical services. Shortages in key specialties, such as anesthesiology and general surgery, can increase the waiting time for surgeries and other invasive procedures [1, 2]. Therefore, it is incumbent on a country’s healthcare leadership to continually examine the physician workforce situation in each medical specialty in order to detect issues that could lead to shortfalls and surpluses and then apply effective corrective actions [3, 4]. This is especially important since healthcare is becoming increasingly complex, populations are growing and the number of elderly patients requiring specialized care is increasing worldwide [5]. However, many countries lack accurate information on the size and composition of their active physician workforce and, thus, lack the formal strategic physician workforce planning programs necessary to implement effective workforce policies [6].

Israel is plagued by shortages of certain specialists, such as pathologists, anesthesiologists and psychiatrists, a situation that regularly affects the efficient functioning of the healthcare system [7, 8]. For example, in 2021, 21 of 34 (62%) anesthesiology department chairs reported that operating rooms were routinely closed or procedures canceled due to shortages of anesthesiologists [9]. Furthermore, the situation is more complicated because the extent of the shortage is affected by geography, with hospitals located in the country’s periphery suffering greater workforce shortages when compared to those in the country’s center [10].

To evaluate the present and future needs of the physician workforce, healthcare leaders have a number of workforce forecasting methods at their disposal [11]. These methods attempt to predict future staffing requirements by using trends based on historical data to estimate the demand for future medical services. However, the accuracy of a forecast is highly dependent on the validity of the prediction method and the accuracy of the variables and data used in its computation. The accuracy of the forecasts is also affected by factors that are either not considered by the forecasting method or non-existent at the time of the forecast.

We previously used data from a 2005 survey of the Israeli anesthesiology workforce to perform needs-based and demand-based predictions to predict the future needs of this workforce [12]. In 2021, a follow-up survey of the existing anesthesiology workforce was performed [9]. The outcome of this follow-up study permits us to compare the previous workforce forecasts to the actual 2021 workforce. Moreover, these comparisons allowed us to assess the usefulness of the different predication methods and to address possible reasons for differences with the data collected 16 years later. The aim was to assess the accuracy of the different predictive methods and identify possible ways of modifying them so as to improve their predictive capabilities. Our assumption was that the accuracy of these workforce predations was primarily affected by unforeseen and disruptive events occurring during the intervening 16 years.

## Methods

Cross-sectional surveys of the Israeli anesthesiology workforce were performed in 2005 (*n* = 711 anesthesiologists in 30 departments) [12] and 2021 (*n* = 1330 anesthesiologists in 36 departments) [9]. In both surveys data were collected from the anesthesiology department chairs (100% response rate to both surveys) about each individual anesthesiologist employed in Israeli public and private hospitals. The assembled information included age, gender, position (resident, certified specialist) and country of medical schooling and anesthesiology residency. The surveys were approved by the Institutional Review Board of the Hadassah Medical Organization. The completion of the survey was considered implied consent.

The data analyses were divided into three phases.

Phase One provided additional information on state of the 2021 anesthesiology workforce. This added data included regional (central vs. peripheral geographic hospitals) differences in the composition of the anesthesiology workforce and changes from 2005 to 2021 in the size of individual hospital anesthesiology departments.

Phase Two compared the needs-based and demand-based forecasts of future anesthesiology workforce requirements originally made using the 2005 survey data with the actual workforce size observed in the 2021 survey. We scrutinized under and over-estimations to ascertain their possible causes. We assessed possible causes for discrepancies between the 2005 forecasts and the results of the 2021 survey that were explored included unanticipated changes in anesthesiology, surgical and procedural practices; changes in anesthesiologists’ working conditions; and demographic issues, such as unanticipated changes in population growth plus the change in the proportion of the population ≥ 65 years. These latter data were obtained from information published by the Israel Central Bureau of Statistics [13]. In addition, the annual volume of specific surgical procedures performed in Israel since 2005 was obtained from publications of the Organization for Economic Co-operation and Development (OECD) and Israel Ministry of Health [14, 15]. These assessments of possible under and overestimations were performed while recognizing that the demand for physician services is also influenced by patients’ socioeconomic status, geographical factors, disease burden, health care system access and insurance coverage.

Phase Three involved assessing the accuracy of the different predictive methods, identifying the reasons that they did not provide accurate forecasts and proposing possible ways of improving their accuracy.

### Data analysis

Needs-based and demand-based workforce forecasting methods used in the 2005 projections were the forecasting methods examined in the present study.

Needs-based forecasting methods, which use physician-to-population ratios (e.g. number of physicians per 100,000 persons), are relatively simple and are thus frequently used. The ratios can either be based on historical data or on desired present and/or future ratios. Workforce projections can then be calculated based on the estimated growth of the population and/or the estimated change in the number of physicians. An important caveat when using this method is that basing future physician workforce requirements on the existing ratio of physicians per capita does not account for unmet current needs, future increases in needs or non-linear increases in population growth.

Demand-based projections use physician-to-service (or utilization; e.g. surgeries, hospital admissions) ratios. This method estimates future requirements (demand growth) based on the current or a future desired level of service and the current and/or future desired number physicians, i.e., this method attempts to predict the size of the workforce required to keep up with the projected demand for services. However, one must note that this method uses health care service/utilization patterns as proxies for patient demand or clinical necessity for services. It does not account for unmet demand.

The needs-based and demand-based workforce projections calculated in 2005 were compared to the actual 2021 workforce in order to assess whether the 2005 predictions were accurate. To provide direct comparisons we specifically compared the data from the 30 departments (*n* = 711 anesthesiologists) surveyed in 2005 to the data from the same 30 departments in 2021 (*n* = 1227 anesthesiologists). If the 2005 projections were not found to be accurate, the reasons were explored, and, where possible, corrections made to the input variables and calculations in an attempt to improve the 2020 projections. Accuracy was defined as the prediction being within ± 20% of the actual number of anesthesiologists in 2021.

The nature of the data distributions of Phase One data, the change in the number of anesthesiologists in the 30 departments surveyed both in 2005 (*n* = 711 anesthesiologists) and again in 2021 (*n* = 1227 anesthesiologists), was explored with the Kolmogorov-Smirnov test. Normally distributed data are presented as mean ± SD. The central tendency of continuous, non-normally distributed data is reported as median and range. The magnitude of the increases in anesthesiologists from 2005 to 2021 in the centrally located public hospitals was compared to the increases in peripherally located ones using two-tailed Students t-tests. Categorical data are reported as relative percentages and compared using Chi-square tests. Best-fit regression analysis was used to assess projected surgical volumes. Multivariable linear regression analysis was used to examine the association of increase in anesthesiology department size with hospital type (public vs. private), size (number of beds) and geographic (central vs. peripheral) location. Public hospitals are non-profit institutions operated by the Ministry of Health, the Clalit Health Maintenance Organization and non-government organizations such as the Hadassah Medical Organization. Private hospitals are for-profit institutions operated by a variety of corporations. The anesthesiology department size was the dependent variable with hospital type, hospital size and geographic location being the independent variables. P values < 0.05 were considered to indicate statistical significance.

## Results

### Phase one

The total number of anesthesiologists in the 30 departments surveyed both in 2005 and again in 2021 increased by 73% (711 to 1227 anesthesiologists). The mean of the individual departmental increases was 98 ± 90% (SD) (median: 80%; range: 0%-367%). The wide range was due to a small public hospital department without an increase while two of three private hospital departments had increases of > 350%. The total number of anesthesiologists in all 27 public hospitals increased by 69% with the mean of the individual department increases being 74 ± 45% (median: 73%; range: 0-200%). The number of anesthesiologists in the seven large tertiary care public hospitals (Rambam Health Care Campus, Haifa; Rabin Medical Center – Belinson Campus, Petach Tikva; Chaim Sheba Medical Center at Tel HaShomer, Tel Hashomer; Sourasky-Ichilov Medical Center, Tel Aviv; Hadassah University Medical Center-Ein Kerem, Jerusalem; Shaare Zedek Medical Center, Jerusalem; Soroka Medical Center, Be’er Sheva) increased by 76% with the mean of the individual department increases being 80 ± 19% (median: 83%; range: 51–106%). Upon multivariable regression analysis, the magnitude of the percentage increase in department size was significantly associated with private ownership (coef = 2.41, *p* < 0.001) and not with hospital size or geographic location. The magnitude of the increases in anesthesiologists from 2005 to 2021 in the centrally located public hospitals did not differ significantly from those in the peripherally located ones (central: 468 to 799: 71%; peripheral: 209 to 343; 64%; NS).

### Phase two

#### Needs-based analysis

**Fig. 1:**
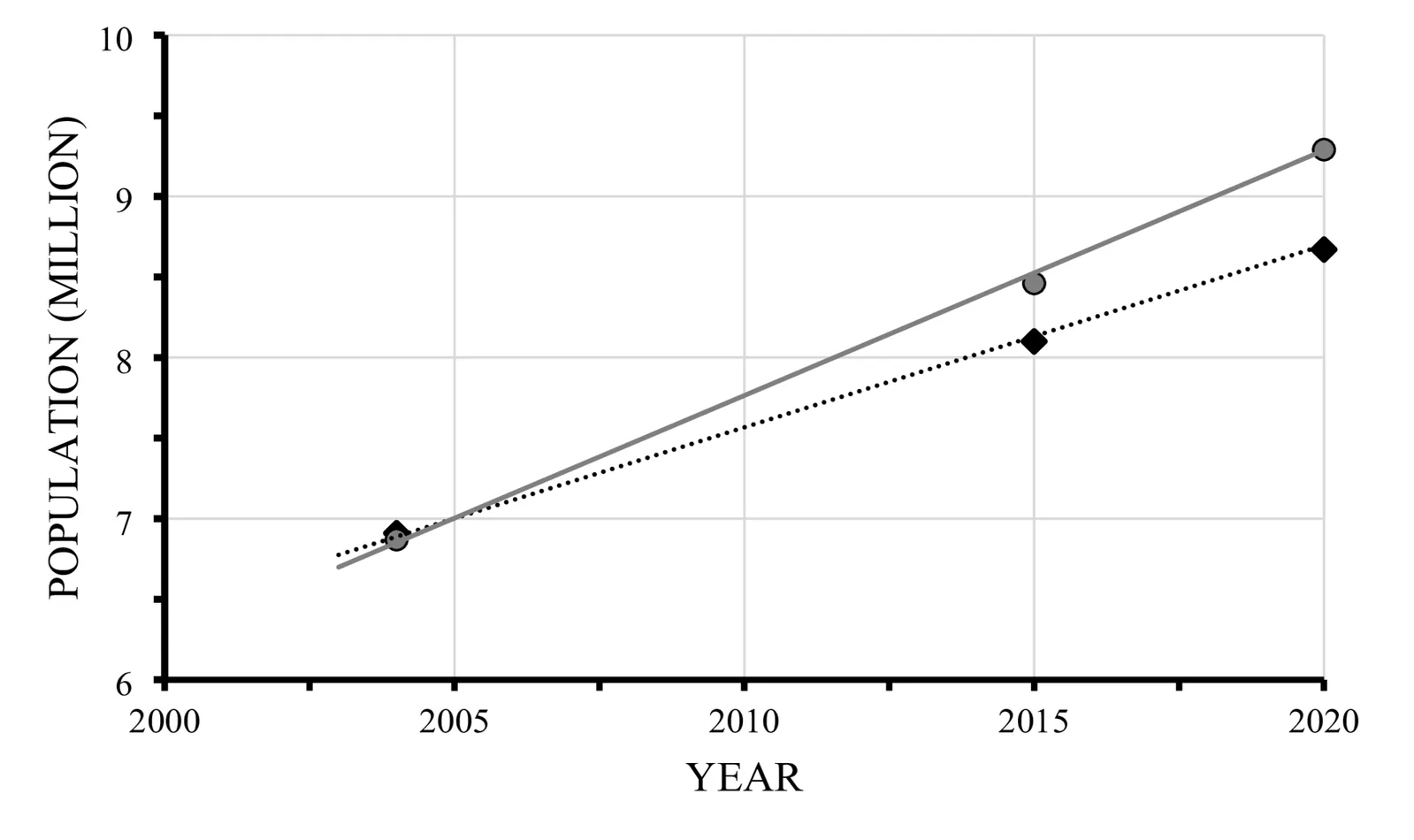
The population estimate used in the 2006 paper (dotted black line) is displayed. Its trendline is y = 0.1131x − 219.68, (R² = 0.999). The actual population increased at a higher rate from 2005 to 2020 (solid gray line) than was estimated. The trendline is y = 0.1522x − 298.11 (R² = 0.998)

The original needs-based analysis based on the 2005 data was used to examine the projected needs for 2021 and demonstrated that they underestimated the actual increase in anesthesiologists (Table 1 - Needs-Based Projections). The projections made in 2005 used estimates of the future growth of the Israeli populations. However, the population grew at a faster rate (Fig. 1). and is one of the reasons that the 2005 predictions underestimated the actual growth in the number of anesthesiologists. The 2005 projections were recalculated using the actual Israeli population and these projections underestimated by 50–54% the actual increase in anesthesiologists (Table 1 - Needs-Based Projections). To provide direct comparisons we specifically compared the data from the 30 departments (*n* = 711 anesthesiologists) surveyed in 2005 to the data from the same 30 departments in 2021 (*n* = 1227 anesthesiologists), the ratio of anesthesiologists to population in 2021 was 13.2 anesthesiologists per 100,000 population, an increase from the 10.8 anesthesiologists per 100,000 population reported in the 2005 survey. Applying the updated ratio to all 36 hospitals (*n* = 1330 anesthesiologists) underestimated the actual total number of anesthesiologists in 2021 reflecting the large number of anesthesiologists in the six remaining hospitals, which aside from the new Assuta-Ashdod Hospital were private hospitals (Table 1 - Needs-Based Projections). When the entire group of 1330 anesthesiologists (36 departments) reported in the 2021 survey was examined the ratio was 14.3 anesthesiologists per 100,000 population (Table 1). This ratio is similar to the ratio in the United Kingdom [16].

**Table 1:** Needs-based projections

Projections	Additional anesthesiologists needed − 2005–2021		
	Assumption #1*	Assumption #2**	Assumption #3***
Original 2006 paper projections based on 10.8 anesthesiologists per 100,000 population and the estimated population increase in 2021	195	232	269
Recalculation of 2006 paper projection based on 10.8 anesthesiologists per 100,000 population and the actual population increase in 2021	261	271	281
Recalculation of 2006 projections using 13.2 anesthesiologists per 100,000 population and the actual population increase in 2021	319	327	336
Recalculation of 2006 projections using 14.3 anesthesiologists per 100,000 population and the actual population increase 2021	346	353	363

* No extra anesthesiologist per department

** One extra anesthesiologist per department

*** Two extra anesthesiologists per department

#### Demand-based analysis

**Fig. 2:**
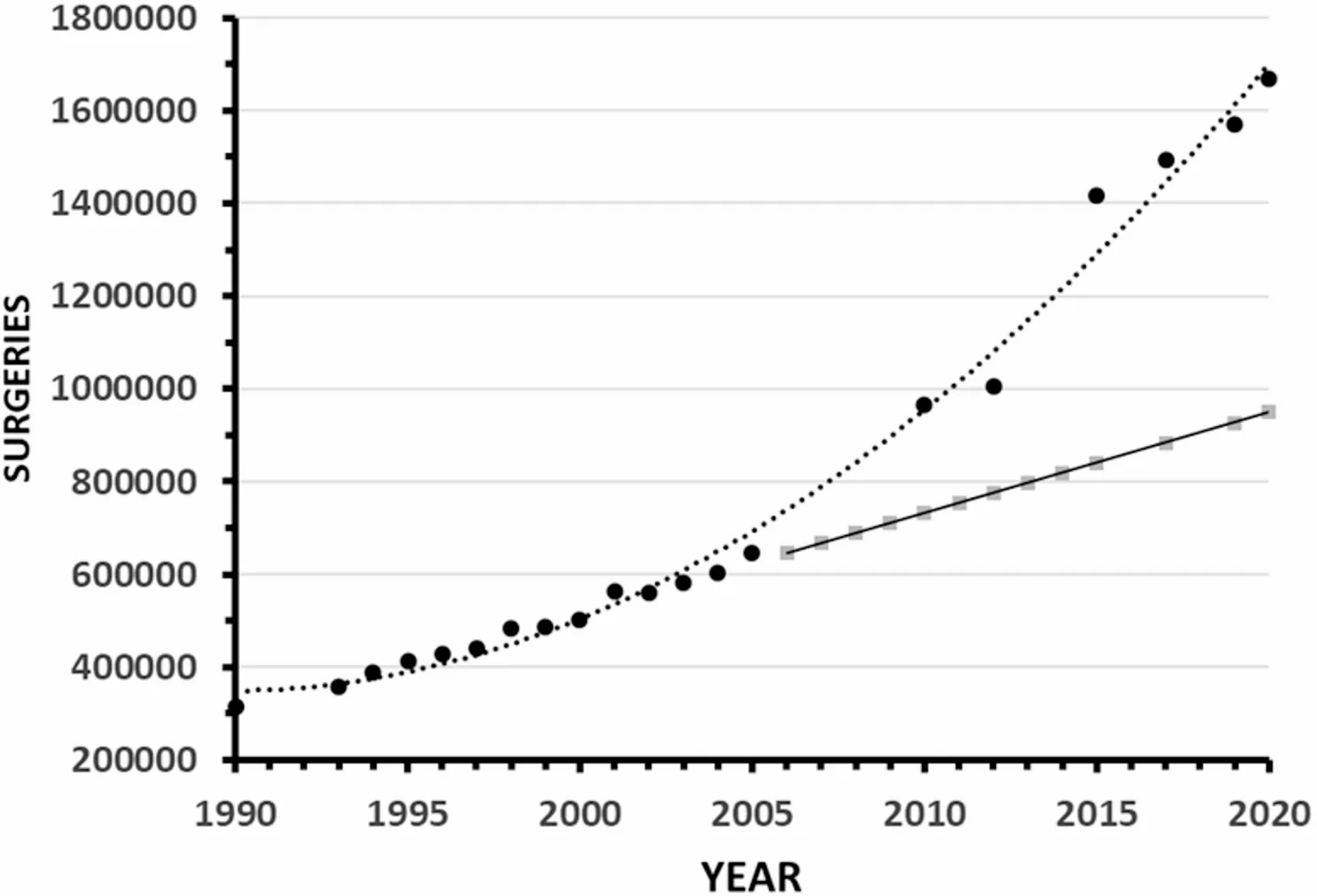
Annual surgical volume: when the total number of procedures reimbursed by the health funds is considered the increase from 1990 to 2020 is curvilinear (dotted black line). The linear extrapolation of the 2005 data is also shown (solid gray line)

**Fig. 3:**
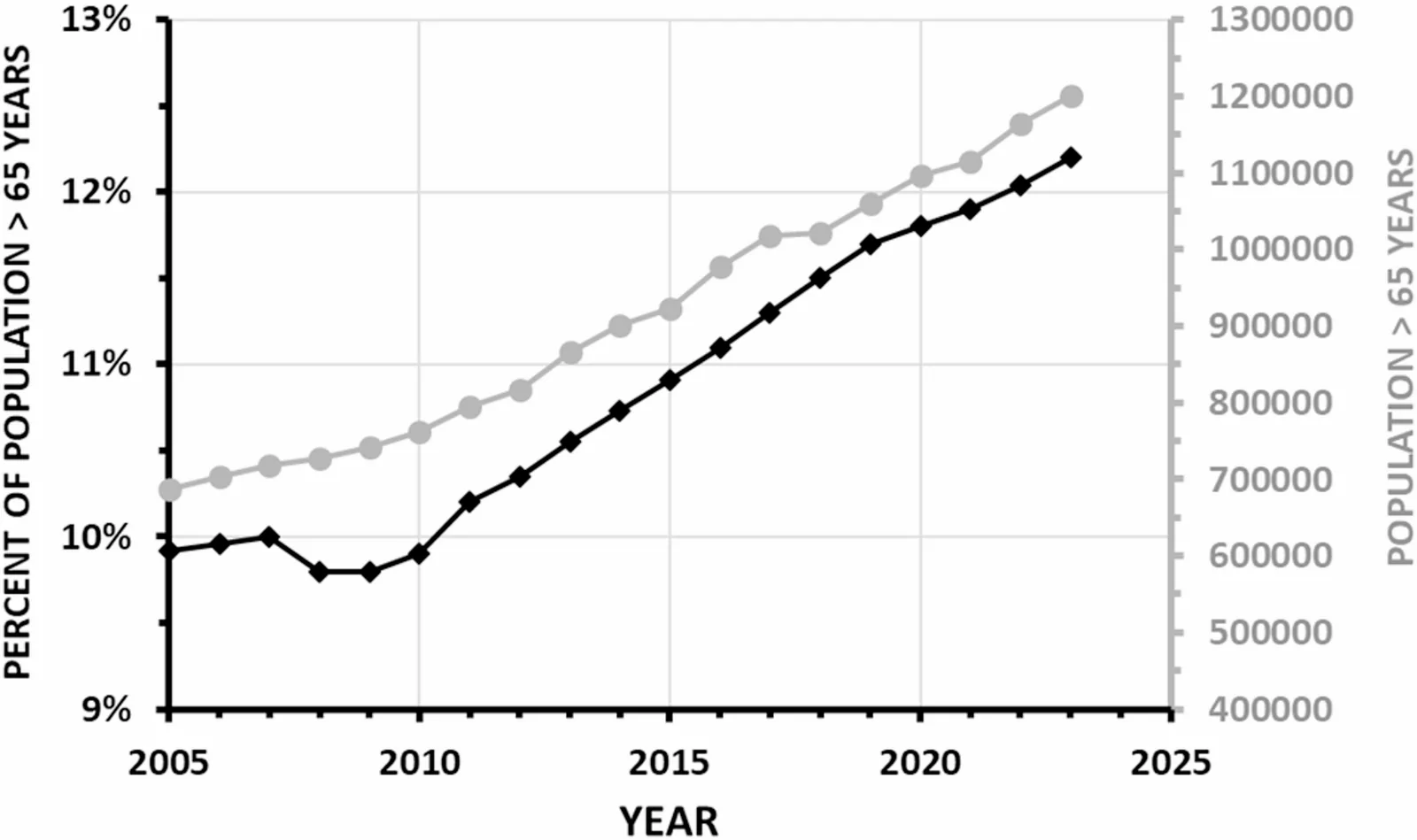
The percent of the Israeli population (black line) and the actual population (gray line) older than 65 years increased continuously over 23 years (2000–2023)

**Fig. 4:**
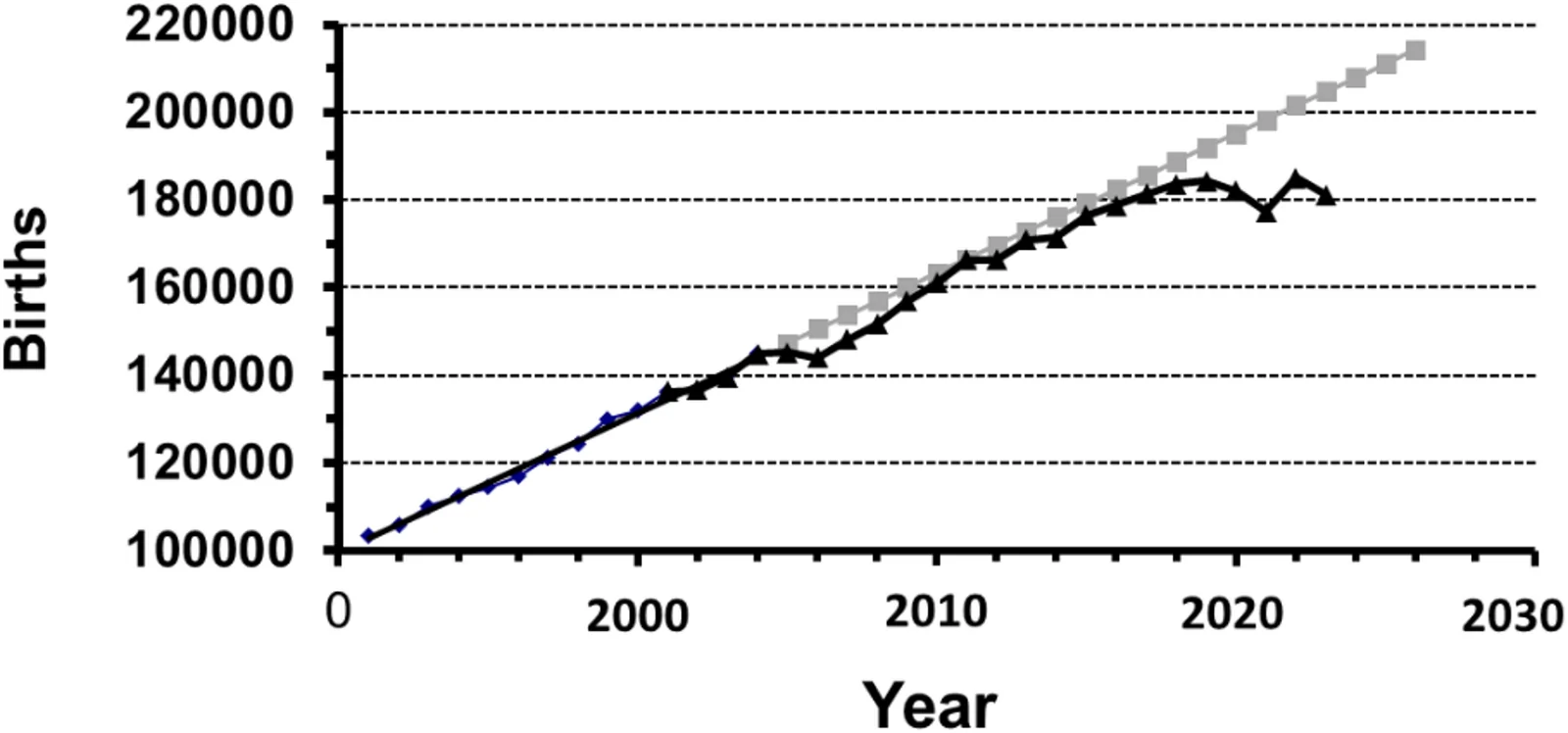
Displayed is the birth rate in Israel (black line) and the predicted increase (y = 3186.6x + 99546; R² = 0.9927) based on pre-2005 data (gray line). In the last three years there were smaller increases in the birth rate

The demand-based projections performed in 2005 also underestimated the actual increase in the 2021 anesthesiology workforce. The data from 2005 projected the need for 267 additional anesthesiologists. Even with the addition of 5% additional anesthesiologists for working outside the anesthesiology department the projection was for 281 additional anesthesiologists. The major reason for this underestimation was that the number of surgeries increased more than anticipated in 2005. Linear models based on older (1995–2005) and newer (2013–2015) Ministry of Health data showed a greater increase in surgeries when using the 2015 data (2005 estimate = 774,442 vs. 2015 model– 840,688; 8.6% difference). Datasets containing different types of surgeries from the OECD [14] and Israel Ministry of Health showed 25% and 12% increases, respectively, when data from 2011 was compared to those from 2019. Data from the Israel State Comptroller’s Office reported a greater exponential increase, although these data are based on the total number of procedures reimbursed by the four health funds and thus includes non-surgical procedures, such as cardiac catheterizations and gastrointestinal endoscopies, many of which do not require the presence of an anesthesiologist. (Fig. 2). Reasons for the significant increase in surgeries appears multifactorial. The population increased at a rate greater than predicted (Fig. 1). The proportion of the population > 65 years old increased from 9.9% in 2005 to 12.1% in 2021 which with the population increase and the increase in life expectancy (2005–80.1; 2021–83.2) resulted in more elderly individuals (Fig. 3). The birth rate also increased requiring a greater number of caesarean sections and epidural catheter insertions for pain control (Fig. 4). The 2005 projections were based on each anesthesiologist performing 879 anesthetics for surgeries per year. However, the exponential data revealed 1063 anesthetics per anesthesiologist per year, an increase of 21%. The linear data revealed that a decrease of 10% to 791 anesthetics for surgeries per year.

## Discussion

The present study shows that forecasting the long-term needs of the Israeli physician anesthesiologist workforce is an endeavor fraught with unpredictable hurdles. The limitations of predictions made 16 years previously patently demonstrated that assumptions based on statistical forecasting require frequent updates to account for both unpredictable and disruptive scenarios. Increases in both the rate of population growth, the increased proportion of elderly inhabitants and changes in surgery and procedure patterns were among the factors that affected the forecasts. These changes, when coupled with the increase in procedures and anesthetics in locations outside the operating rooms, made relying only on the number of surgeries performed in operating rooms problematic. Furthermore, disruptive changes in working conditions due to renewed and revised union contracts affected workforce needs, as did the opening of a new public hospital (Samson Assuta Ashdod Medical Center, Ashdod Israel). In addition, the expansion of activity in private hospitals necessitated their employing many more anesthesiologists. Moreover, a major finding of the present study was that the actual workforce was significantly greater than what the 2005 forecasts had predicted would be needed in 2021. However, even the 2021 workforce was insufficient to meet the healthcare system’s needs as evidenced by over 60% of anesthesiology department chairs reporting experiencing workforce shortages within their departments [9].

### Anesthesiology department workforce changes − 2005–2021

From 2005 to 2021, there was a median increase of 80% (2005 − 711 anesthesiologists; 2021 − 1227 anesthesiologists) in the number of anesthesiologists in the 30 hospital anesthesiology departments included in the 2005 survey, with greater increases seen in private hospitals. This latter observation reflects the increasing proportion of surgical care being provided in the private sector, especially since the reforms (in 2018) that permitted private for-profit hospitals to become providers of publicly-funded procedures [17]. This resulted in these hospitals hiring more full-time anesthesiologists rather than partially relying on moonlighters from the public sector. Although we found that in the private sector the increase in the number of anesthesiologists was associated with the increase in the number of beds, it is important to note that in such hospitals the bed count is not necessarily a precise indicator of extent their surgical activity due to the considerable ambulatory surgery activity. These observations further explain the expansion of the anesthesiology workforce over 15 years.

### Sources of forecast error

#### Needs-based projections

Many healthcare systems adopt the principle that the supply of healthcare services should be based on their population’s need for care and thus use needs-based forecasting methods [18]. In the present study, simple needs-based projections which rely solely upon projected population changes proved problematic. The main limitation of this forecasting method was its underestimating future needs because the Israeli population increased at a rate greater than anticipated in 2005. However, there were other reasons for the needs-based projections underestimating the need for additional anesthesiologists:

1. Simple needs-based projections do not account for unmet healthcare needs, which if present, result in underestimations of actual workforce needs [19]. In both 2005 and 2021 the majority of anesthesiology department chairs reported a shortage of anesthesiologists which affected their ability to meet demand [12, 9]. Therefore, the ratio of 10.8 anesthesiologist per 100,000 population observed in 2005 and 13.2 anesthesiologist per 100,000 population found in 2021 were both insufficient to meet the demand for anesthesia services.
2. The simple needs-based projection underestimated demand for additional anesthesiologists by omitting key determinants of healthcare workforce needs. These include greater demand for anesthesia services out of proportion to the increase in the population. This increased demand was due to an increase in the proportion of the population > 65 years old and an increased birth rate requiring a greater number of caesarean sections. This is outlined in more detail in the section on demand-based projections. These are examples of the limitations of simple needs- based projections that are based on assumptions that the morbidity within the population does not change [18]. However, morbidity increases as the proportion of elderly inhabitants increases resulting in a greater need for medical services and thus more anesthesiologists per 100,000 population [18, 20]. Therefore, more complex needs-based forecasting methods add variables such as age and sex, disease burden (per age and sex) and the extent of health service interventions needed to treat common health conditions [21, 22]. Other factors often included in upgraded needs-based projection methods include the retirement of medical staff and hours worked within given age and sex groups [23].
3. Another reason for the underestimation was expansion of anesthesia services outside the operating rooms. For example, the interventional neuroradiology market increased from 1.89 billion dollars in 2017 to 2.28 billion dollars in 2021 [24].
4. The Israel Medical Association – Ministry of Health union contract in 2011 included an additional 2 weeks of vacation for each anesthesiologist (“radiation workers”) plus addition post-call time off (an additional day off for a Friday 8:00am to Saturday 8:00am shift). These disruptive changes, unanticipated in 2005, resulted in the need for additional anesthesiologists but did not increase clinical services, i.e. they reduced physician productivity. Moreover, productivity is a factor that needs to be incorporated into workforce models, especially, since work–life balance is a prominent issue among younger physicians who wish to work fewer hours [25].

These unanticipated and disruptive changes demonstrate the many shortcomings of simple needs-based projections especially when the projections are 16 years old and when the original forecasts are based on a workforce with existing shortages.

#### Demand-based projections

The demand-based projections were plagued by the lack of comprehensive publicly available data. The available data did not include current information on the total annual number of surgeries performed. These data were often incomplete, including only some types of surgeries, and then only for a limited number of years [14]. This required using indirect techniques to estimate surgical activity. In addition, there were no data available on anesthetics performed outside the operating rooms. This lack of information is not surprising since, unlike the operating rooms, many of these locations are not equipped with computerized anesthesia charting systems.

The demand-based projections based on the 2005 data found that each anesthesiologist performed 879 anesthetics per year. Linear regression estimates of the number of surgeries performed in operating rooms per year derived from older Ministry of Health data revealed a decrease of 10% to 791 anesthetics per year in 2021. This reduction may be explained as a combination of the changes in anesthetic practice from 2005 to 2021 with more procedures being done outside the operating rooms as well as anesthesiologists having an additional two weeks of vacation time due to the 2011 union contract. The latter reduces anesthesiologist availability by 4–5% per year. Additionally, the exponential data revealed 1063 anesthetics per anesthesiologist per year based on all the surgeries and procedures reimbursed by the health funds including those performed outside the operating room. However, these data did not differentiate between those involving an anesthesiologist and those that did not, and, thus failed to provide data specifically about anesthesiologists’ activity.

Over the 16 years between the two surveys of the Israeli anesthesiology workforce there have been changes in surgical and procedural practices that need to be considered when using demand-based forecasting, especially anesthetics performed outside the operating rooms. For example, there is an increasing demand for anesthesiologists to sedate and anesthetize children outside the operating rooms. A multi-center report from the United States revealed that 23% of 2,236,788 pediatric anesthetics (age < 18 years` 2015–2019) occurred in non-operating room settings, such as the endoscopy and MRI suites [26]. These patients were more complex (higher ASA Physical Status Classification Scores) than those encountered in the operating rooms. Another example is open cardiac procedures that have been partially superseded by percutaneous procedures performed in cardiac catheterization suites, only some with the presence of an anesthesiologist. Israeli OECD data showed a decrease from 2010 to 2022 of 26% in the number of coronary bypass graft surgeries (43.6 to 32.1 per 100,000 persons; an 8% absolute decrease in cases) and an increase of 2% (257.8 to 263.0 per 100,000 persons; a 28% absolute increase in cases) in the number of transluminal coronary artery angioplasty procedures [14]. Ministry of Health data showed no growth in cardiac surgeries from 2011 to 2019. Furthermore, the percent of aortic valve replacements for isolated aortic stenosis performed transcutaneously increased from 45% in 2015–2016 to 88% in 2022, due to fewer open surgical aortic valve replacements [27, 28]. When transcutaneous aortic valve replacements and mitral valve repairs are performed in cardiac catheterization suites an anesthesiologist is generally present. Some patients undergoing these procedures are older and frailer and would not be considered candidates for open surgical procedures [29]. In Israel, the number of transcutaneous aortic, mitral and tricuspid procedures continues to increase [30].

Like the changes in the cardiac arena, more vascular surgery procedures, such as abdominal aortic aneurysm replacements, are performed endovascularly rather than surgically. These are performed in hybrid operating rooms within the operating room area or interventional radiology suites outside the operating rooms. Procedures formerly performed in operating rooms, such as insertion of venous access catheters and ports are often performed in the interventional radiology suite, some with the presence of an anesthesiologist, especially in pediatric patients. Yet at the same time some operating room-based surgeries increased. During the study period, both in Israel and the United States, knee and hip replacements increased due to an aging and, increasingly obese population, coupled with advances in joint prosthesis technology [31, 32, 33]. Similarly, the aging male population fueled an increase in prostate surgeries.

### Implications for modeling

**Fig. 5:**
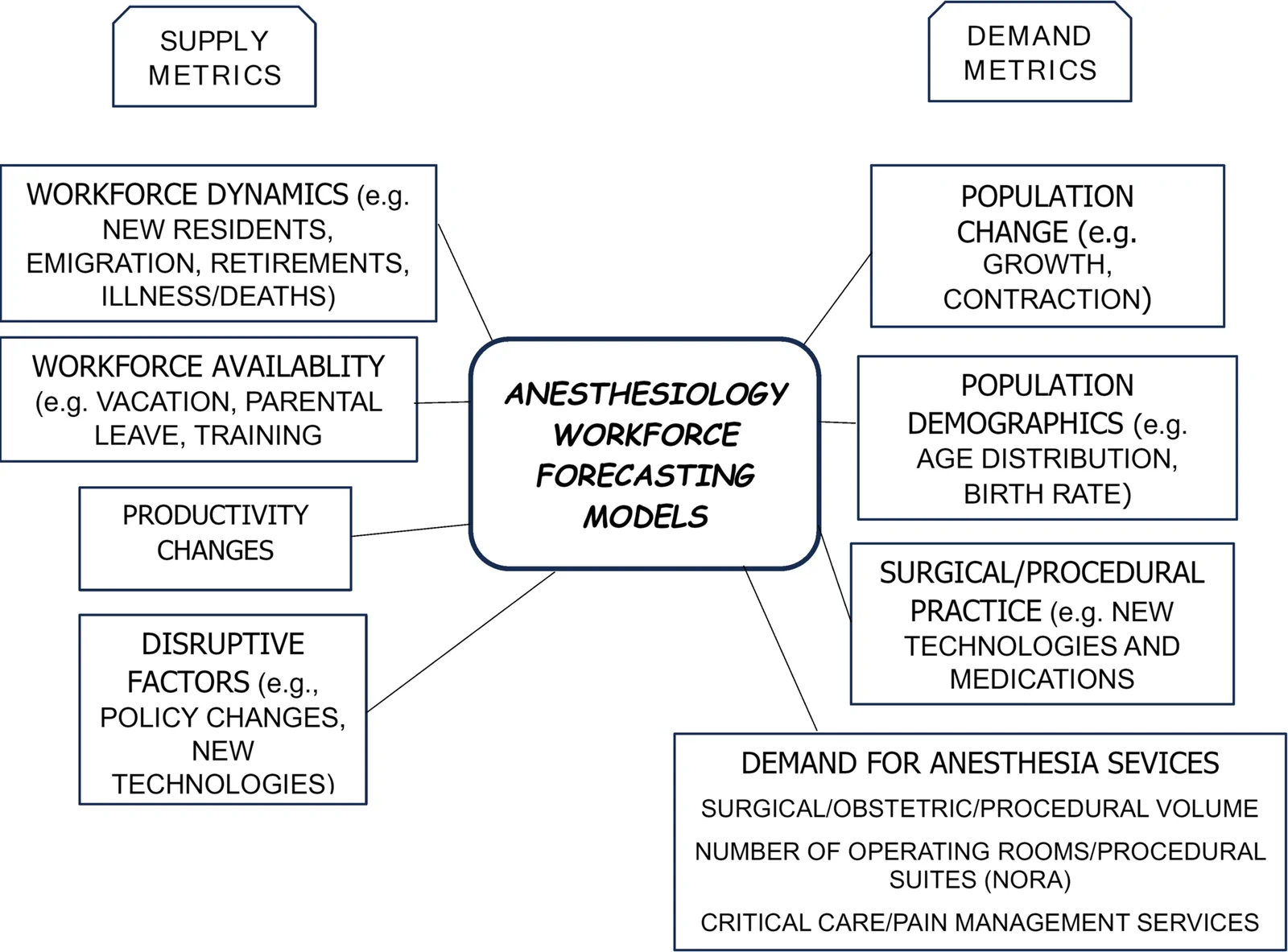
Factors to consider in workforce forecasting models. NORA – Non-Operating Room Anesthesia

There are many factors to consider when forecasting future physician workforce (Fig. 5). Demand models should include additional variables to assess future demand for surgery, such as demographics (e.g. age, sex, ethnicity), health-related lifestyle indicators (e.g. obesity, smoking), health conditions (e.g. cardiovascular disease, diabetes, hypertension) and socioeconomic status (household annual income) [34]. In addition, the decrease in open surgeries in favor of longer duration minimally invasive ones (e.g. laparoscopic and robotic surgeries) requires more anesthesia time per surgery [35, 36, 37, 38]. These newer surgical techniques thus reduce the number of cases per day that anesthesiologists can anesthetize resulting in the needs for both more operating rooms and more anesthesiologists.

### Policy implications

Comparing the accuracy of anesthesiology workforce projections, especially demand-based projections, made in 2005 to the workforce realities 16 years later revealed data collection and availability issues that need resolution. Governmental agencies (Ministry of Health, Central Bureau of Statistics) should collect and make freely available, on an ongoing basis, accurate and validated information on the number and types of surgeries and procedures performed in hospitals and ambulatory surgery centers, their location (e.g. operating room, endoscopy suite, interventional radiology suite, cardiac catheterization suite) and whether an anesthesiologist was present. Such data would be even more robust if it included the total (anesthesia + surgery) duration of the surgical/procedural encounter. This information should include data from the labor and delivery suites, including cesarean sections and epidural catheter insertions for labor pain management. Such data should facilitate increasing the accuracy of demand-based predictions A challenge for demand-based prediction methods is that using utilization data to assess the demand for healthcare services can be biased by inappropriately high or low utilization rates. The latter are due to lack of accessibility to healthcare services, such as in rural areas, and by lack of services, such as in underserved areas [18]. Ultimately, policymakers must optimize workforce adequacy, which is defined as the ratio of physician supply over patient demand for healthcare. This optimization should be performed for each medical specialty and subspecialty [39].

### Policy implications – long-term planning

The results of the present study should be an impetus for the healthcare system to establish a robust long-term workforce planning organization that uses more sophisticated tools rather than the simple needs and demand-based methods we used previously. This organization also should simultaneously use multiple forecasting tools to provide inter-method comparisons. Moreover, this organization should continually monitor supply (workforce) and demand (workload) realities by examining poorly met (long waiting times) and unmet demands not only for surgical and procedural services but cognitive ones too. Furthermore, projections must consider the lag time (medical school and residency) until an anesthesiologist or any other specialist is fully trained. Projection must also take into consideration the continual advances in surgical and procedural practices. Projections of the workforce needs 7, 12 and 15 years in the future are important since these timeframes align with the duration of medical school and residency. Policies that result from these projections should be comprehensive by covering the entire continuum, from medical school acceptance, residency and initial employment. For example, a policy that increases the number of medical students but does not provide sufficient residency positions encourages emigration and prevents the return of Israeli citizen who studied medicine abroad. Another issue to examine is the number of entering anesthesiology residents and the optimal ratio of residents to senior anesthesiologists. The increased complexity of surgeries and procedures (both in and outside the operating rooms) along with an aging population requires training anesthesia residents to handle complex procedures and complicated patients. This will require more operating rooms to have a ratio of 1 senior anesthesiologist to 1 anesthesia residents rather than the more common 1 senior anesthesiologist to 2 anesthesia residents. Moreover, in the non-operating room setting training residents in a ratio of 1 senior anesthesiologist to 1 anesthesia residents is usually required. Additionally, a policy that does not provide sufficient post-residency jobs also encourages emigration and also dissuades the return of Israeli citizen doing post-residency fellowships abroad. Another issue that needs to be addressed is the effect of the new and disruptive on-call model gradually being implemented in Israel. In this model both senior and resident anesthesiologists only begin their night shift in the afternoon thus requiring additional anesthesiologists to cover their morning shift duties. Policies should further address sufficiently staffing short-staffed specialties and limiting overstaffing in oversubscribed ones.

### Policy recommendations – data analytics

Data analytics, the science of analyzing large amounts of raw data to derive descriptive, prescriptive and predictive information, is used by many service and consumer organizations to better understand their business activities and provide an objective basis for planning changes and improvements [40]. Many modern healthcare systems have implemented electronic medical and hospital record (EMR, EHR) systems for routine use. These large information systems incorporate large databases containing much administrative and clinical data. Therefore, they should be able to provide continual objective data on the number (workforce) and activities (workload) of anesthesiologists both in and outside the operating rooms to agencies, such as the Ministry of Health and the Scientific Council of the Israel Medical Association, responsible for planning the healthcare workforce [41]. To ensure the accuracy of this information, internal and external data retrospective reconciliation exercises along with comparisons with other data sources will be required, similar to those performed to reconcile Israeli national quality indicator data [42]. This information should provide data about the size and clinical activities of the existing physician workforce and its workload, including information on the volume, locations and types of anesthetic services provided [43]. Moreover, because such data could be obtained continually (quarterly or semi-annually) they should facilitate frequent updated forecasts of future workforce needs. For example, information about each anesthesiologist and their annual workload could provide productivity data which can help with demand-based predictions. This continual ability to “mine” and analyze actual data should also enable frequent examination of short and long-term trends. This should overcome some of the limitations of the intermittent predictions such as performed in the current study. Furthermore, in order to accurately assess anesthesiology workload, such data must provide information on anesthetics and sedations performed by anesthesiologists outside the operating rooms and include data from labor and delivery rooms and other locations. These data must be combined with up-to-date demographic and other data, such as population growth, population > 65 years and waiting time for elective surgeries to help provide accurate and timely forecasts of future physician workforce and workload needs. This should help improve the accuracy of needs-based forecasts. Therefore, healthcare system leaders and policy makers should allocate research and development resources to develop the capabilities to accurately capture and then analyze workforce and workload data found in hospital and other healthcare organization information systems. Once developed, up-to-date forecasts calculated using continually improved and robust methods need to be made available to healthcare system leaders and policy makers. Furthermore, these analyses should help design even more robust methods that track and forecast the physician workforce and associated fiscal needs (44).

### Strengths and limitations

Old forecasts are like old news - soon forgotten - and pundits are almost never asked to reconcile what they said with what actually happened. (Attributed to Philip Tetlock)

This study’s strength is that the “pundits” have reconciled their workforce projections to the actual workforce 16 years later and have demonstrated that “it’s tough to make predictions, especially about the future”. Specifically, it demonstrates the many factors that interfere with workforce predictions including those that are easily tracked, such as accelerated population grow, and those which are totally disruptive and unforeseen, such as the provisions of a new union contract. It also showed the problems of obtaining accurate data on the surgical, procedural and anesthesiology activity in Israel. A major limitation of the study is the lack of accurate publicly available data that complicated comparing the 2005 demand-based predictions with the 2021 realities. However, these difficulties provide the healthcare system leadership and health services research community with the challenge to collect and retrospectively reconcile such data, so that demand-based predictions improve in accuracy and their accuracy can be properly evaluated years later. Another limitation is that the needs and demand-based forecast methods used are simple and do not account for variables such as the aging of the population. Future predictions thus should be more sophisticated by incorporating additional variables.

## Conclusions

Never make predictions, especially about the future. (Variously attributed to Niels Bohr, Yogi Berra and Casey Stengel)

Retrospectively reconciliating the results of previous anesthesiologist workforce predictions with the actual workforce needs 16 years later, pointed out the limitations of relying on long and medium-range forecasts. Among the limitations were the many factors that can influence both workforce needs and supply, ranging from unanticipated changes in demographics to disruptions secondary to labor contracts to changes in surgical/procedural practices. Israeli and other healthcare leaders should thus support both the development of more robust forecasting methods that utilize real-time digital tools and frequent re-reconciliation of these forecasts using up-to-date data. This reconciliation should aid in improving the robustness of the forecasting methods so that it is possible to “make predictions, especially about the future.”

## Data Availability

All data generated or analyzed during this study are included in this published article.
